# Prognostic value of the distance of proximal resection margin in patients who have undergone curative gastric cancer surgery

**DOI:** 10.1186/1477-7819-12-296

**Published:** 2014-09-23

**Authors:** HaengJin Ohe, Woo Yong Lee, Seong Woo Hong, Yeo Goo Chang, Byungmoo Lee

**Affiliations:** Department of Memorial Jin Pok Kim Gastric Cancer Center, Inje university Paik Hospital, Inje University College of Medicine, 9, Mareunnae-ro, Jung-gu, Seoul, 100-032 Korea; Department of Surgery, Inje university Paik Hospital, Inje University College of Medicine, 9, Mareunnae-ro, Jung-gu, Seoul, 100-032 Korea

**Keywords:** Gastric cancer, Proximal resection margin (PRM), Locoregional recurrence

## Abstract

**Background:**

We conducted this retrospective study to analyze the relationship between the distance of the proximal resection margin (PRM) and the pattern of recurrence in patients with gastric cancer who underwent curative gastrectomy.

**Methods:**

In our series, there were 774 patients who underwent curative gastrectomy for gastric adenocarcinoma. Thus, we classified our clinical series of patients into the distal gastrectomy group (n = 529) and the total gastrectomy group (n = 245). The clinical pathologic data and PRM distance were collected. Univariate and multivariate analyses were performed to evaluate association between PRM distance and locoregional recurrence.

**Results:**

The mean distance of the PRM was 4.03 cm in the total gastrectomy group. The distance of the PRM had a significant correlation with advanced T-stage, advanced N-stage,vascular invasion,lymphatic invasion, neural invasion, histological undifferentiation, greater tumor size, and the upper third of the tumor location. On multivariate analysis, tumor recurrence showed only the independent prognostic factor N-stage (*P* <0.023). The mean distance of the PRM was 6.4 cm in the distal gastrectomy group. The distance of the PRM had a significant correlation with the advanced T-stage, advanced N-stage, younger age, vascular invasion, histological undifferentiation, greater tumor size, and the middle third of tumor location. On multivariate analysis, tumor recurrence showed three independent prognostic factors, N-stage (*P* <0.001), vascular invasion (*P* = 0.009), and lower third tumor location (*P* = 0.035). The total gastrectomy of locoregional recurrence was related to N-stage (*P* = 0.039), and the distal gastrectomy of locoregional recurrence was related to T-stage (*P* = 0.021). Study on the disease-free survival, PRM distance, and locoregional recurrence was not statistically relevant in both the total and distal gastrectomy group (*P* = 0.565 and *P* = 0.584, respectively).

**Conclusions:**

Our results indicate that a sufficient resection margin is not the absolute factor associated with the rate of survival and recurrence, although it is a key prognostic factor. The locoregional recurrence had no significant correlation with the distance of the PRM after curative gastrectomy.

## Background

Gastric cancer is the fourth most prevalent malignancy in Western countries, and its incidence is the highest in Korea [1, 2]. It is widely accepted that surgery is the first line of therapy in patients with gastric cancer, for whom curative marginal resection is the only method for increasing the survival rate. Curative gastric cancer surgery basically includes stomach resection, lymph node dissection, and reconstruction. In patients with gastric cancer, surgical interventions are routinely performed considering both their conditions and cancer characteristics. To date, active studies have been conducted to identify prognostic factors in these patients. Thus, the pathologic condition after radical surgical resection has been established as the key prognostic factor. Both the lack of invasion of cancer cells at the resection margin and the appropriate dissection of surrounding lymph nodes are essential for obtaining successful treatment outcomes of curative resection [3].

It is mostly recommended that the resection margin be remote from the margin of the tumor for the prevention of its recurrence. In association with this, several studies have reported that the inadequate resection margin is related to locoregional recurrence [4–6]. Currently, however, there are no established guidelines for the distance of the resection margin; it is routinely subject to surgeons’ technical expertise. The first part of the duodenum, 1 to 2 cm distal to the pylorus, is routinely used as the distal resection margin (DRM) in gastrectomy. However, the distance of the proximal resection margin (PRM) remains controversial, despite the fact that it is a key factor that is associated with the extent of gastrectomy.

We conducted this retrospective study to analyze the relationship between the distance of the PRM and the pattern of recurrence in patients with gastric cancer who underwent curative gastrectomy.

## Methods

### Study population

We performed surgical operations for a total of 896 patients with gastric adenocarcinoma at department of Memorial Jin Pok Kim, Korea Gastric Cancer Center, Inje Medical College, Seoul Paik Hospital, Seoul, Korea between September 2002 and December 2006. Of these, 122 patients were excluded because of other synchronous or metachronous cancers, positive surgical margins, or palliative treatment for gastric cancer. Therefore, 774 patients who were treated with curative gastrectomy were enrolled in the current study. Of these, 529 who underwent distal gastrectomy (29 underwent Billroth I surgery and 500 underwent Billroth II surgery) and 245 who underwent total gastrectomy. Thus, our clinical series of patients were classified into the distal gastrectomy group (n = 529) and the total gastrectomy group (n = 245). Types of surgical procedure were determined by the attending surgeon’s preference, mainly based on the gastric cancer treatment guidelines of Japan [3].

Patients’ clinical pathological data was collected from medical and computerized records. Based on pathologic examination, we retrospectively analyzed the clinical stage, T- and N-stage, tumor size, histologic type, Lauren’s classification, venous, lymphatic, and neural invasion, tumor location, and the distance of the PRM. The distance of the PRM was defined as the distance extending from the proximal limit of the lesion to the site of the resection. It was not consistent with the intraoperative measurement. It was measured based on the results obtained from surgical specimens placed in 10% formalin solution for more than 12 hours. It was routinely evaluated using an intraoperative frozen section biopsy in all the patients. We analyzed the relationship between various clinicopathological factors and the recurrence, including the locoregional recurrence in particular.

The study population included patients who underwent radical gastrectomy with D2 lymphadenectomy. We made a diagnosis of cancer recurrence on physical examination, radiologic assessment, and endoscopy. Recurrences were classified as locoregional recurrence, hematogenous metastasis, peritoneal carcinomatosis, and multiple metastases. Main patterns of recurrence were recorded as the first site of detection at the time of diagnosis. By definition, the locoregional recurrences include local lymph node metastasis, extraluminal recurrence, recurrence within the gastric remnant stomach, and anastomotic recurrence after gastrectomy. The tumor stage was reported according to the sixth edition of the tumor-node-metastasis (TNM) classification of malignant tumor established by the International Union Against Cancer (UICC) [7]. Our clinical series of patients were followed up on until December 2011, with a mean follow-up period of 45 months (range: 1 to 111 months).

We obtained approval for this study from the Institutional Review Board at Inje university Paik Hospital. Written informed consent was obtained from patient for the publication of this report and any accompanying images.

### Statistical analysis

Statistical analysis was done using SPSS version 12.0 for Windows (SPSS, Inc., Chicago, Illinois, United States). All data was expressed as mean ± standard definition (SD). A *P*-value of <0.05 was considered to be statistically significant. Independent sample Student’s t-tests were used to analyze the age, tumor size, and the distance of the PRM. In addition, the chi-squared test was used to analyze the differences in the above variables between the two groups. Furthermore, a log-rank test (univariate analysis) with the Kaplan-Meier method was used to analyze the disease-free survival (DFS) and the distance of the PRM. The Cox proportional hazards model and logistic regression tests were employed for multivariate analysis.

## Results

### Baseline and demographic data of the patients

In our series, there were 529 patients who underwent distal gastrectomy and 245 who underwent total gastrectomy. Thus, we classified our clinical series of patients into the distal gastrectomy group (n = 529) and the total gastrectomy group (n = 245). The mean age of the patients was 60 years (range: 24 to 88 years) in the distal gastrectomy group and 60 years (range: 24 to 87 years) in the total gastrectomy. The male-to-female ratio was 1.3:1.0 in the total gastrectomy group and 3.5:1.0 in the distal gastrectomy group. In both groups, sex and age had no significant correlation with the distance of the PRM, with the exception of age in the distal gastrectomy group. It is likely, early cancer is probably more, statistical significance was observed, that there is no clinical significance.

In both groups, the sex had no significant correlation with the distance of the PRM. But, age in the distal gastrectomy group was correlated with PRM diastnace. It is likely, early cancer is many subtota gastrectomy group, it was observed a statistically significant, but it seems not to be clinically significant.

### The distance of the proximal resection margin in the total gastrectomy group

The mean distance of the PRM was 4.03 cm in the total gastrectomy group. That is, there were 68 (27.8%), 73 (29.8%), and 104 (42.4%) patients where the distance of the PRM was <2 cm, 2 to 4 cm, and >4 cm, respectively. The distance of the PRM had a significant correlation with advanced T-stage (*P* <0.001), advanced N-stage (*P* <0.001), vascular invasion (*P* = 0.007), lymphatic invasion (*P* = 0.003), neural invasion (*P* <0.001), histological undifferentiation (*P* = 0.045), greater tumor size (*P* <0.001), and the upper third of the tumor location (*P* <0.001) (Table 1).

**Table 1:** **Clinicopathological factors depending on the distance of the proximal resection margin (PRM) in the patients who underwent curative total gastrectomy**

Parameter	PRM (cm)				***P*** value
	≤2	2 - 4	≥4		
**Gender**					0.305
Male	35	42	62	139	
Female	33	31	42	106	
**Age (years)**					0.403
≤60	28	35	50	113	
>60	40	38	54	132	
**T-stage**					<0.001
T1	2	21	36	59	
T2	24	17	37	78	
T3	40	28	29	97	
T4	2	7	2	11	
**N-stage**					<0.001
N0	11	27	52	90	
N1	14	19	23	56	
N2	13	8	10	31	
N3	30	19	19	68	
**Vascular invasion**					0.007
Absent	37	54	78	169	
Present	31	19	26	76	
**Lymphatic invasion**					0.003
Absent	7	23	33	63	
Present	31	19	71	182	
**Neural invasion**					<0.001
Absent	6	28	37	71	
Present	62	45	67	174	
**Histology**					0.045
Differentiated	13	18	34	65	
Undifferentiated	55	55	70	180	
**Lauren’s classification**					0.316
Intestinal	9	16	28	53	
Diffuse	43	42	49	134	
Mixed	16	15	27	58	
**Tumor size (cm)**					<0.001
≤2	0	6	13	19	
2 - 5	8	26	43	77	
>5	60	41	48	149	
**Tumor location**					<0.001
Lower third	0	1	13	14	
Middle third	13	34	73	120	
Upper third	34	31	18	83	
Entire	21	7	0	28	

There were 81 patients who had a tumor recurrence; there were 16 cases of locoregional recurrence, 41 cases of peritoneal carcinomatosis, 11 cases of hematogenous metastasis, and 13 cases of multiple metastases. In addition, the recurrence was found to have a significant correlation with T-stage (*P* <0.001), N-stage (*P* <0.001), PRM (*P* = 0.032), vascular invasion (*P* <0.001), lymphatic invasion (*P* <0.001), neural invasion (*P* <0.001), histology (*P* = 0.004), Lauren’s classification (*P* = 0.006), tumor size (*P* <0.001), and tumor location (*P* <0.001) (Table 2). Multivariate analysis performed by applying logistic regression showed only a significant correlation with N-stage (*P* <0.023).

**Table 2:** **Correlations with clinicopathological factors, recurrence, and locoregional recurrence in the patients who underwent curative total gastrectomy**

Parameter	Recurrence		***P*** value	Locoregional recurrence		***P*** value
	Negative	Positive		Negative	Positive	
**Gender**			0.592			<0.001
Male	95	44		29	15	
Female	69	37		36	1	
**Age (years)**			0.655			0.869
≤60	74	39		31	8	
>60	90	42		34	8	
**T-stage**			<0.001			0.917
T1	59	0		0	0	
T2	62	16		13	3	
T3	39	58		46	12	
T4	4	7		6	1	
**N-stage**			<0.001			0.013
N0	83	7		7	0	
N1	42	14		7	7	
N2	13	18		15	3	
N3	26	42		36	6	
**Vascular invasion**			<0.001			0.400
Absent	131	38		32	6	
Present	33	43		33	10	
**Lymphatic invasion**			<0.001			0.309
Absent	59	4		4	0	
Present	105	77		61	16	
**Neural invasion**			<0.001			0.844
Absent	65	6		5	1	
Present	99	75		60	15	
**Histology**			0.004			0.771
Differentiated	53	12		10	2	
Undifferentiated	111	69		55	14	
**Lauren’s classification**			0.006			0.021
Intestinal	45	8		6	2	
Diffuse	81	53		47	6	
Mixed	38	20		12	8	
**Proximal resection margin (cm)**			0.032			0.910
≤2	37	31		25	6	
2 - 4	51	22		17	5	
≥4	76	28		23	5	
**Tumor size (cm)**			<0.001			0.771
≤2	19	0		0	0	
2 - 5	65	12		10	2	
>5	80	69		55	14	
**Tumor location**			<0.001			0.122
Lower third	7	7		4	3	
Middle third	83	37		32	5	
Upper third	64	19		13	6	
Entire	10	18		16	2	

On univariate analysis, the pattern of recurrence had a significant correlation with locoregional recurrence after curative resection; these include gender (*P* <0.001), N-stage (*P* = 0.013) and Lauren’s classification (*P* <0.021) (Table 2). Multivariate analysis showed that the N-stage was the only independent prognostic variable associated with the locoregional recurrence (*P* = 0.039). The risk of locoregional recurrence in patients with N3 was 2.677-fold higher than that in patients with other N-stages (hazard ratio: 3.484; 95% CI: 1.053 to 6.805).

**Figure 1:**
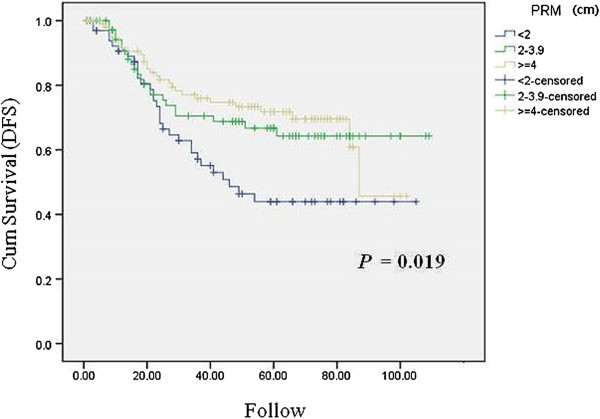
**Correlations between the distance of the PRM and recurrence in patients who underwent curative total gastrectomy.** Kaplan-Meier curves for cumulative DFS with total recurrence according to the distance of the PRM. DFS, disease-free survival, PRM, proximal resection margin. The distance of the proximal resection margin in the distal gastrectomy group.

On univariate analysis based on the Kaplan-Meier method, the cumulative DFS was significantly shorter in the patients with a shorter distance of the PRM as compared with those with a longer distance of the PRM (*P* = 0.019) (Figure 1). However, there was no significant correlation between the cumulative locoregional recurrence and the PRM (*P* = 0.565). Ultimately, while the PRM distance in total gastrectomy influenced the DFS, it had no effect on the locoregional recurrence.

The mean distance of the PRM was 6.4 cm in the distal gastrectomy group. That is, there were 71 (13.4%), 187 (35.3%), and 271 (51.2%) patients where the distance of the PRM was <3 cm, 3 to 6 cm, and >6 cm, respectively. The distance of the PRM had a significant correlation with advanced T-stage (*P* <0.001), advanced N-stage (*P* <0.001), younger age (*P* = 0.044), vascular invasion (*P* = 0.013), histological undifferentiation (*P* <0.001), greater tumor size (*P* <0.001), and the middle third of the tumor location (*P* <0.001) (Table 3).

**Table 3:** **Clinicopathological factors depending on the distance of the proximal resection margin (PRM) in the patients who underwent curative distal gastrectomy**

Parameter	PRM (cm)				***P*** value
	≤3	3 - 6	≥6		
**Gender**					0.936
Male	47	138	189	374	
Female	24	49	82	106	
**Age (years)**					0.044
≤60	42	86	119	247	
>60	29	101	152	282	
**T-stage**					<0.001
T1	29	97	181	307	
T2	23	47	65	135	
T3	18	42	24	84	
T4	1	1	1	3	
**N-stage**					<0.001
N0	37	106	187	330	
N1	14	42	60	116	
N2	9	22	18	49	
N3	11	17	6	34	
**Vascular invasion**					0.013
Absent	59	158	248	465	
Present	12	29	23	64	
**Lymphatic invasion**					0.057
Absent	29	90	144	263	
Present	42	97	127	266	
**Neural invasion**					0.062
Absent	39	108	176	323	
Present	32	79	95	206	
**Histology**					<0.001
Differentiated	20	67	146	233	
Undifferentiated	51	120	125	296	
**Lauren’s classification**					0.199
Intestinal	24	66	127	217	
Diffuse	33	81	81	195	
Mixed	14	40	63	117	
**Tumor size (cm)**					<0.001
≤3	25	68	151	244	
3 - 5	14	46	60	120	
≥5	32	73	60	165	
**Tumor location**					<0.001
Lower third	33	87	217	337	
Middle third	38	100	54	192	

There were 73 patients who had a tumor recurrence; there were 26 cases of locoregional recurrence, 19 cases of peritoneal carcinomatosis, 17 cases of hematogenous metastasis, and 11 cases of multiple metastases. In addition, the recurrence had a significant correlation with T-stage (*P* <0.001), N-stage (*P* <0.001), vascular invasion (*P* <0.001), lymphatic invasion (*P* <0.001), neural invasion (*P* <0.001), tumor size (*P* <0.001), and tumor location (*P* = 0.003) (Table 4). Multivariate analysis performed by applying logistic regression showed that the recurrence had a significant correlation with N-stage (*P* <0.001), vascular invasion (*P* = 0.009), and the lower third of tumor location (*P* = 0.035). On univariate analysis, the pattern of recurrence had no significant correlation with locoregional recurrence after curative resection (Table 4). Multivariate analysis showed that the T-stage was the only independent prognostic variable associated with the locoregional recurrence (*P* = 0.021); the risk of locoregional recurrence in T3-stage patients was 16.308-fold higher than that in patients with other T-stage classifications (hazard ratio: 3.484; 95% CI: 1.535 to 173.245).

**Table 4:** **Correlations with clinicopathological factors, recurrence, and locoregional recurrence in the patients who underwent curative distal gastrectomy**

Parameter	Recurrence		***P*** value	Locoregional recurrence		***P*** value
	Negative	Positive		Negative	Positive	
**Gender**			0.348			0.172
Male	319	55		33	22	
Female	137	18		14	4	
**Age (years)**			0.302			0.100
≤60	217	30		16	14	
>60	239	43		31	12	
**T-stage**			<0.001			0.599
T1	301	6		3	3	
T2	111	24		14	10	
T3	42	42		29	13	
T4	2	1		1	0	
**N-stage**			<0.001			0.555
N0	326	4		2	2	
N1	95	21		14	7	
N2	26	23		17	6	
N3	9	25		14	11	
**Vascular invasion**			<0.001			0.957
Absent	426	39		25	14	
Present	30	34		22	12	
**Lymphatic invasion**			<0.001			0.682
Absent	256	7		5	2	
Present	200	66		42	24	
**Neural invasion**			<0.001			0.975
Absent	306	17		11	6	
Present	150	56		36	20	
**Histology**			0.118			0.520
Differentiated	207	26		18	8	
Undifferentiated	249	47		29	18	
**Lauren’s classification**			0.356			0.275
Intestinal	192	25		18	7	
Diffuse	167	28		19	9	
Mixed	97	20		10	10	
**Proximal resection margin (cm)**			0.054			0.275
≤3	55	16		10	6	
3 - 6	161	26		14	12	
≥6	240	31		23	8	
**Tumor size (cm)**			<0.001			0.862
≤3	240	4		3	1	
3 - 5	102	18		12	6	
≥5	114	51		32	19	
**Location**			0.003			0.108
Lower third	279	58		40	18	
Middle third	177	15		7	8	

**Figure 2:**
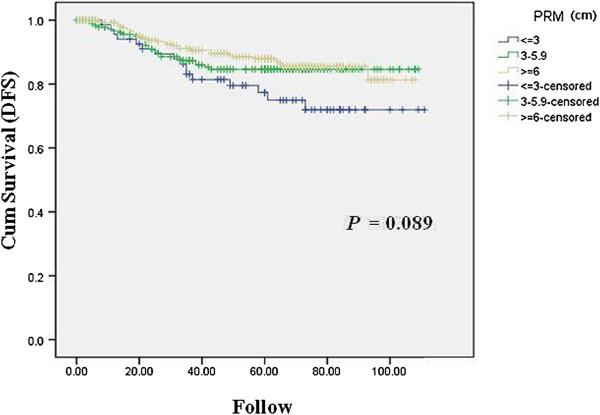
**Correlations between the distance of the PRM and recurrence in the patients who underwent curative distal gastrectomy.** Kaplan-Meier curves for cumulative DFS with total recurrence according to the distance of the PRM. DFS, disease-free survival, PRM, proximal resection margin.

On univariate analysis using the Kaplan-Meier method, the cumulative DFS had no significant correlation with the distance of the PRM (*P* = 0.089) (Figure 2). Also, there was no significant correlation between the cumulative locoregional recurrence and the PRM (*P* = 0.584).

## Discussion

In the past, total gastrectomy was considered as a standard modality in patients with gastric cancer because it could safely assure marginal distance [8, 9]. However, there were no significant differences in the survival rate and complications between the subtotal and total gastrectomy in patients where the gastric cancer occurred in the distal third of the stomach [8]. For successful curative gastrectomy, obtaining appropriate resection margin is crucial. The resection margin is determined according to the degree of progression, pathologic findings, location of cancer, and the chance of metastasis to lymph node. The distal margin of resection is usually 1 to 2 cm away from the pylorus, the first part of duodenum. In contrast, the distance of the PRM for gastrectomy remains controversial. It is mostly recommended, however, that the distance of the PRM be set. This is because a positive margin is an independent unfavorable factor for patients who undergo gastrectomy.

According to Bozzetti *et al*. [4], the distance of the PRM should be maintained at 3 cm in patients with gastric cancer without serosal invasion, and >6 cm in those with serosal invasion, or those for whom an accurate assessment is not possible. According to this study, the most important factor affecting cancer infiltration at resection margin is the primary tumor depth of the gastric wall. It is statistically significant, regardless of cancer location, size, gross findings, pathologic findings, and lymph node positivity. According to the Japanese Gastric Cancer Association (JGCA) guidelines, the distance of the PRM should be 1 cm in patients with mucosal invasion, 3 cm in those with well-differentiated muscle layer and serosa, and >5 cm in those with histological undifferentiation and serosal invasion [3]. Further, another report stated that the length of cancer invasion along the gastric wall was several millimeters at the margin of cancer lesion on gross findings in patients with intestinal type gastric cancer, and several centimeters in those with diffuse type gastric cancer [5]. Consistent with this, Gall and Hermanek [10] reported that the length of cancer invasion was >8 cm in patients with diffuse type gastric cancer and >4 cm in patients with intestinal type gastric cancer. Moreover, Ha and Kwon [5] reported that the survival rate was significantly higher in patients with advanced gastric cancer located in the distal third who had a >3-cm resection margin. In the current study, however, there was no significant difference in survival rate between patients with advanced gastric cancer located in the middle third and proximal third who had a >3-cm resection margin, and those who had a <3-cm resection margin. Moreover, our results also showed that the distance of the PRM had a significant correlation with the advanced T-stage, advanced N-stage, vascular invasion, lymphatic invasion, neural invasion, histological undifferentiation, greater tumor size, and the upper third of tumor location in patients who underwent total gastrectomy. However, the recurrence had a significant correlation with N-stage only. The distance of the PRM had a significant correlation with advanced T-stage, advanced N-stage, vascular invasion, lymphatic invasion, neural invasion, histological undifferentiation, greater tumor size, and the middle third of tumor location in patients who underwent distal gastrectomy. However, the recurrence had a significant correlation with N-stage, vascular invasion, and the lower third of tumor location.

It is generally known that there are three major patterns of recurrence in patients with gastric cancer undergoing curative surgery; these include locoregional recurrence, peritoneal dissemination, and distant metastasis. The locoregional recurrence is the most common in Western countries, but is rare in Asian countries [11, 12]. From this context, Sasako *et al*. [13] confirmed that peritoneal dissemination was the main recurrence type of gastric cancer after curative gastrectomy with an extended lymphadenectomy according to the JCOG (Japan Clinical Oncology Group) study 9501. In the current study, we found that peritoneal dissemination was the most common, followed by the multiple recurrences in the patients who underwent total gastrectomy. In the patients who underwent distal gastrectomy, however, locoregional recurrence was the most common, followed by peritoneal dissemination.

Deng *et al*. [14] analyzed 169 cases of recurrence in patients with gastric cancer who were treated with curative resection, reporting that there was a significant correlation between the locoregional recurrence and Lauren’s classification. Moreover, Yoo *et al*. [15] analyzed 508 cases of recurrence in patients with gastric cancer who were treated with potentially curative gastrectomy, reporting that 33% of total recurrences involved the locoregional site. These authors also noted that the locoregional recurrence had a significant correlation with older age, large tumor size, diffuse type, and proximal location. According to other studies, there are clinicopathologic factors with a predictive value for the locoregional recurrence and these include the proximal location, older age, male sex, advanced stage (T3 to 4, and Nodal metastasis), infiltrative growth, diffuse type, and stromal resection [12, 16, 17]. In our clinical series of patients, on univariate analysis, the male sex, N-stage, and Lauren’s classification had a significant correlation with the locoregional recurrence after curative total gastrectomy. On multivariate analysis, however, the N-stage was the only independent prognostic factor that was found to be associated with the locoregional recurrence. In patients who underwent curative distal gastrectomy, however, no significant correlation with locoregional recurrence was found on either univariate or multivariate analysis.

It is generally recommended that the length of resection margin be sufficient for lowering the chance of local recurrence, but this does not apply to all cases. Papachristou and Fortner argued that at least a 6.5-cm resection margin should be maintained to prevent local recurrence [6]. In addition, Nakamura *et al*. [18] insisted that cancer invasion at the resection margin is not frequent and meaningless in patients with early gastric cancer, although it is a key prognostic factor. Consistent with this, Kim *et al*. [19] reported that the distance of the PRM had no negative effects on the prognosis of patients with early gastric cancer. According to these authors, there was a close relationship between the distance of the PRM and the prognosis in patients with advanced gastric cancer, but they contradicted the relationship between the distance of the PRM and locoregional recurrence. Our results show that there was no significant correlation between the locoregional recurrence and the distance of the PRM.

## Conclusions

In conclusion, our results indicate that a sufficient resection margin is not the absolute factor associated with the rate of survival and recurrence, although it is a key prognostic factor. The locoregional recurrence had no significant correlation with the distance of the PRM after curative gastrectomy. The distance of the PRM observed in the operative field or the range of palpation is important, but it should be carefully evaluated as microscopic tumor may be present depending on the characteristics of the tumor. It would therefore be mandatory to intraoperatively perform a frozen section biopsy of the PRM. In addition, it is also necessary to avoid excessive resection in patients who are negative for an intraoperative frozen section biopsy of the PRM, which is essential for improving the quality of life and minimizing the occurrence of complications in patients undergoing curative gastrectomy.

## Abbreviations

DFS: Disease free survival
DRM: Distal resection margin
PRM: Proximal resection margin
TNM: Tumor-node-metastasis
UICC: Union for International Cancer Control.

## References

[1] Korean Gastric Cancer Association (2002). Nationwide gastric cancer report in Korea. J Korean Gastric Cancer Assoc.

[2] Ferlay J, Bray F, Pisani P, Parkin DM (2004). GLOBOCAN 2002: Cancer Incidence, Mortality And Prevalence Worldwide.

[3] Nakajima T (2002). Gastric cancer treatment guidelines in Japan. Gastric Cancer.

[4] Bozzetti F, Bonfanti G, Bufalino R, Menotti V, Persano S, Andreola S, Doci R, Gennari L (1982). Adequacy of margins of resection in gastrectomy for cancer. Ann Surg.

[5] Ha TK, Kwon SJ (2006). Clinical importance of the resection margin distance in gastric cancer patients. J Korean Gastric Cancer Assoc.

[6] Papachristou DN, Fortner JG (1981). Local recurrence of gastric adenocarcinomas after gastrectomy. J Surg Oncol.

[7] Hermanek P (1982). Surgical pathology–the TNM system. Langen-becks Arch Chir.

[8] Gouzi JL, Huguier M, Fagniez PL, Launois B, Flamant Y, Lacaine F, Paquet JC, Hay JM (1989). Total versus subtotal gastrectomy for adenocarcinoma of ther gastric antrum. A French prospective controlled study. Ann Surg.

[9] Bozzetti F, Marubini E, Bonfanti G, Miceli R, Piano C, Gennari L, The Italian Gastrointestinal Tumor Study Group (1999). Subtotal versus total gastrectomy for gastric cancer. Ann Surg.

[10] Gall FP, Hermanek P (1985). New aspects in the surgical treatment of gastric carcinoma-a comparative study of 1,636 patients operated on between 1969 and 1982. Eur J Surg Oncol.

[11] Roviello F, Marrelli D, de Manzoni G, Morqaqni P, Saraqoni L, De Stefano A (2003). Prospective study of peritoneal recurrence after curative surgery for gastric cancer. Br J Surg.

[12] Maehara Y, Hasuda S, Koga T, Tokunaga E, Kakeji Y, Sugimachi K (2000). Postoperative outcome and sites of recurrence in patients of following curative resection of gastric cancer. Br J Surg.

[13] Sasako M, Sano T, Yamamoto S, Kurokawa Y, Nashimoto A, Kurita A, Hiratsuka M, Tsujinaka T, Kinoshita T, Arai K, Yamamura Y, Okajima K (2008). D2 lymphadenectomy alone or with para-aortic nodal dissection for gastric cancer. N Engl J Med.

[14] Deng JG, Liang H, Wang DC, Sun D, Pan Y, Liu Y (2011). Investigation of the recurrence patterns of gastric cancer following a curative resection. Surg Today.

[15] Yoo CH, Noh SH, Shin DW, Choi SH, Min JS (2000). Recurrence following curative resection for gastric carcinoma. Br J Surg.

[16] Wu CW, Lo SS, Shen KH, Hsieh MC, Chen JH, Chiang JH, Lin HJ, Li AY, Lui WY (2003). Incidence and factors associated with recurrence patterns after intended curative surgery for gastric cancer. World J Surg.

[17] Schwarz RE, Zagala-Nevarez K (2002). Recurrence patterns after radical gastrectomy for gastric cancer: prognostic factors and implications for postoperative adjuvant therapy. Ann Surg Oncol.

[18] Nakamura K, Ueyama T, Yao T, Xuan ZX, Ambe K, Adachi Y, Yakeishi Y, Matsukuma A, Enjoji M (1992). Pathology and prognosis of gastric carcinoma: findings in 10,000 patients who underwent primary gastrectomy. Cancer.

[19] Kim SH, Karpeh MS, Klimstra DS, Leunq D, Brennan MF (1999). Effect of microscopic resection line disease on gastric cancer survival. J Gastrointest Surg.

